# A Guide to Constructing Indigenous Statistical Spaces for Prevention Science Research

**DOI:** 10.1007/s11121-026-01911-5

**Published:** 2026-05-08

**Authors:** Valentín Quiroz de la Sierra

**Affiliations:** Center for Indigenous Health, Department of International Health, Bloomberg School of Public Health, Johns Hopkins University, 415 N. Washington St., Baltimore, MD 21231 USA

**Keywords:** Prevention science, Artificial intelligence, Indigenous Research Methodologies, Deaths of despair, Indigenous Data Sovereignty

## Abstract

Artificial intelligence (AI)-powered computational methods, such as machine learning and natural language processing, are increasingly applied in deaths of despair research among Indigenous populations. However, their application in Indigenous contexts is often constrained by epistemological misalignment, technical limitations, and ethical concerns. Integrating Indigenous Research Methodologies into AI-powered prevention science research is necessary to support Indigenous Data Sovereignty and address deaths of despair. The Indigenous Computational Approach (ICA) provides a structured reflexive protocol for constructing Indigenous Statistical Spaces that operationalize Indigenous Research Methodologies within computational workflows. ICA aligns four interdependent components: Researcher Standpoint, Indigenous Theoretical Frameworks, AI Data Analysis Technique, and Dissemination and Indigenous Governance. This protocol is supported by operational steps and an accompanying ICA Checklist. A previously published case study on the Indigenous Wholistic Factors Project illustrates the ICA in practice in the context of suicide risk modeling. The case study applied a lasso logistic regression model to structure feature selection on an Indigenous subsample of the 2019–2020 California Healthy Kids Survey (*n* = 2609). Ten of 17 candidate features were retained, and the model demonstrated strong discrimination (AUC = 0.87) and acceptable calibration (Brier score = 0.10). The ICA does not guarantee different empirical findings or superior model accuracy, but rather it restructures how AI models are designed, validated, and deployed for prevention science research. The ICA provides a replicable protocol for AI-powered prevention science research to support Indigenous self-determination and community-defined well-being.

**Supplementary Information:**

The online version contains supplementary material available at https://doi.org/10.1007/s11121-026-01911-5.

## Introduction

Deaths of despair—including suicide, drug overdoses, and alcohol-related mortality—disproportionately impact Indigenous populations in the USA (Friedman & Hansen, 2024; Sterling & Platt, 2022). These deaths are rooted in long-standing economic inequities, historical trauma, and chronic underinvestment in Tribal healthcare systems (Akee et al., 2024; Beals et al., 2005; Ehlers et al., 2022; Gameon & Skewes, 2021). Suicide rates remain nearly twice the national average for Indigenous populations (27.1 vs. 14.2 per 100,000 in 2023), with young adults aged 18 to 29 experiencing sharp increases between 2018 and 2023 (CDC: Centers for Disease Control & Prevention, 2024). Parallel trends in opioid- and alcohol-related mortality further intensify this crisis (Ivanich et al., 2021; Karaye et al., 2023). Addressing deaths of despair requires prevention strategies that reflect social and Indigenous determinants of health (Friedman & Hansen, 2024; Oré et al., 2024).

Prevention science offers a suite of frameworks for identifying risk factors and implementing preventive interventions (Rehder et al., 2021). However, conventional prevention frameworks often treat risk as an individualized behavioral phenomenon and fail to account for Indigenous conceptions of well-being (Allen et al., 2022; Oré et al., 2024; Wexler & Gone, 2012, 2016). This misalignment results in prevention strategies that underemphasize protective factors, such as cultural identity and intergenerational connectedness, which are associated with lower rates of suicide and substance use (Henson et al., 2017; Wiglesworth et al., 2022; Woods et al., 2022). Evidence from culturally grounded interventions and a growing set of Indigenous-led programs, including Healing the Canoe (Donovan et al., 2015) and the Celebrating Life Suicide Prevention Program (Cwik et al., 2016), demonstrate that integrating Indigenous Research Methodologies and community-driven processes produce effective and culturally resonant prevention outcomes (Pham et al., 2021; Richer & Roddy, 2023).

### Artificial Intelligence in Prevention Science Research and Its Failures in Indigenous Contexts

Artificial intelligence (AI)-powered computational methods, such as machine learning and natural language processing, are increasingly used to identify and predict risk factors associated with deaths of despair, particularly in suicide and substance use preventive interventions (Atmakuru et al., 2025; Chhetri et al., 2023). AI models are powerful because they can analyze large datasets, identify latent patterns, and inform targeted interventions for individuals at heightened risk of self-harm or substance misuse (Afshar et al., 2022; Ehtemam et al., 2024; Lo-Ciganic et al., 2019). However, three structural challenges emerge when applying AI-powered prevention science research in Indigenous contexts: (1) epistemological misalignment, where biomedical theories of risk overshadow Indigenous determinants of health; (2) technical limitations, created when AI models rely on data structures that prioritize individual-level clinical features or omit predictive features relevant to Indigenous populations; and (3) ethical concerns, arising from the absence of Indigenous governance over AI model design, validation, and deployment.

Evidence across multiple studies shows that conventional AI models can underperform for Indigenous populations due to epistemological challenges like the misalignment with or exclusion of Indigenous determinants of health (Haroz et al., 2020, 2023, 2024). This is compounded by technical challenges like cross-population domain shift, as one of aspect dataset shift, where a model trained on one specific population’s data encounters a different input distribution when deployed in a new population (Coley et al., 2021; Finlayson et al., 2021). For example, widely deployed suicide prediction models trained on non-Indigenous clinical data can misclassify Indigenous suicide risk by prioritizing individual-level clinical features while overlooking Indigenous determinants of health like cultural identity, intergenerational connectedness, and engagement with traditional healing practices (Dickerson et al., 2022; Donovan et al., 2015; Haroz et al., 2021; Ullrich, 2019). Recent work on developing an Indian Health Service-specific suicide risk model showed that electronic health record-based machine learning can achieve strong discriminatory performance. However, because these models rely on clinical records, they omit Indigenous-specific risk and protective factors that are not captured in electronic health records (Adams et al., 2024). As Adams and colleagues note (2024), these omissions limit what clinical prediction models can represent about Indigenous health and the features that shape suicide risk.

These shortcomings reflect deeper epistemological and technical constraints of healthcare data. AI models often depend on general population data, such as electronic healthcare records, which can exclude care pathways relevant to Indigenous communities such as traditional healing practices and community-based interventions (Haroz et al., 2024). As a result, conventional AI models can risk reduced effectiveness in Tribal healthcare settings by overlooking predictive features relevant to Indigenous populations and encoding only biomedical interpretations of risk.

In addition to technical and epistemological challenges, the deployment of AI models in Indigenous contexts raises serious ethical issues around governance. AI models used in Tribal settings are often developed without Tribal oversight which can perpetuate extractive research practices and misalignment with Indigenous Data Sovereignty principles (Carroll et al., 2020). Reine et al. (2017) define Indigenous Data Sovereignty as the “the right of Indigenous peoples and tribes to govern the collection, ownership, and application of their own data.” Haroz et al. (2023) highlight that the enactment of Indigenous Data Sovereignty through Tribal and community oversight are vital for effective AI model deployment in Tribal healthcare settings. Without mechanisms for Indigenous communities to govern AI model design, validation, and deployment, these AI technologies risk reinforcing colonial dynamics rather than promoting Indigenous self-determination in prevention science research (Rainie et al., 2017).

Together, these epistemological, technical, and ethical challenges demonstrate that AI models cannot be simply transferred into Indigenous contexts without intentional restructuring. While conventional prevention science frameworks offer broad guidance on intervention design, they typically lack methodological tools for integrating Indigenous approaches to research and governance structures into the development of these interventions (Williams & Shipley, 2023). This extends to the design, validation, and deployment of AI models in prevention science research. Similarly, emerging AI fairness frameworks emphasize bias detection and model transparency, but they lack mechanisms for the application of Indigenous Data Sovereignty Principles (Carroll et al., 2020; Roberts & Montoya, 2023). There is therefore a methodological gap between what prevention science offers, what AI fairness frameworks evaluate, and what Indigenous communities require to ensure that AI-powered computational methods reflect Indigenous worldviews and support Indigenous self-determination.

### Indigenous Research Methodologies as the Foundation for Culturally Responsive AI

Indigenous Research Methodologies offer one pathway toward resolving these epistemological, technical, and ethical challenges. Indigenous Research Methodologies structure the research process around Indigenous ways of being, knowing, and doing (Kovach, 2021; Smith, 2021). These frameworks treat research as a way of being structured within the Four R’s of Respect, Reciprocity, Responsibility, and Relationships which define how researchers relate to people, place, and knowledge itself (Kirkness & Barnhardt, 1991). As ways of knowing, research is often guided by Indigenous Knowledge Systems that locate well-being within the interconnectedness of physical, mental, spiritual, and environmental health (Absolon, 2010; Hart, 2010; Smith, 2021). As ways of doing, Indigenous Research Methodologies prioritize relational accountability including community-engaged and participatory processes that center community voices to ensure that knowledge production serves Indigenous self-determination (Hearod et al., 2019; Hudson et al., 2023; Kovach, 2021). Evidence from Indigenous-led prevention programs that incorporate Indigenous Research Methodologies can improve acceptability, effectiveness, and sustainability (Cwik et al., 2016; Donovan et al., 2015; Haroz et al., 2023).

Yet, work is needed to apply Indigenous Research Methodologies to the computation workflow of designing, validating, and deploying of AI models. These methodologies provide a conceptual and ethical foundation but can benefit from an operational protocol that guides researchers in how to shape data structures, select features, interpret model outputs, and govern the deployment of AI models in prevention science research. This paper seeks to address this by providing a replicable and structured reflexive protocol that operationalizes Indigenous Research Methodologies for the unique demands of AI workflows in Indigenous contexts.

## Indigenous Computational Approach

**Fig. 1:**
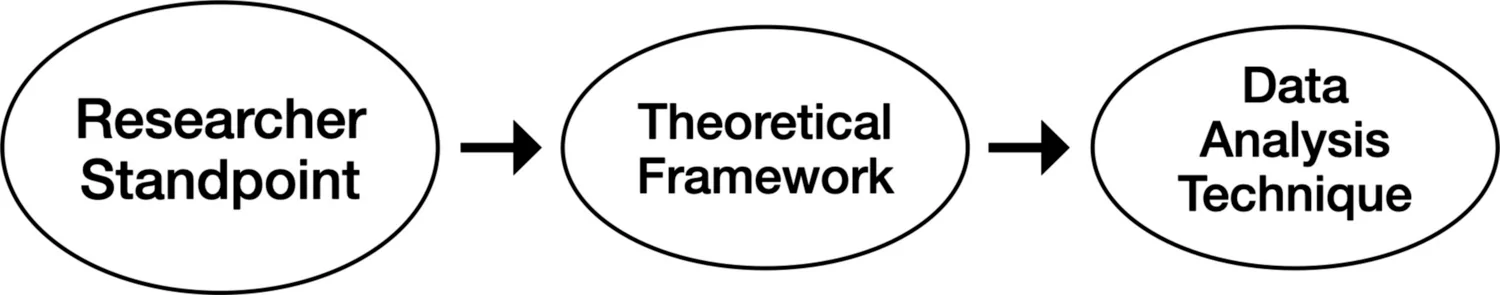
Adapted conceptualization of the three components for constructing Indigenous Statistical Spaces

The Indigenous Computational Approach (ICA) is a structured reflexive protocol for constructing Indigenous Statistical Spaces that integrate AI-powered computation methods with Indigenous Research Methodologies. Indigenous Statistical Spaces function as quantitative research environments where researcher standpoint, theoretical framework, and data analysis technique are deliberately aligned to honor Indigenous governance systems and community-defined priorities (Walter & Andersen, 2013; see Fig. 1). Rather than adapting AI models after the fact, the ICA restructures the computational workflow itself from the beginning by asking how researchers enter the work, how models are conceptually framed, and how data and algorithms are configured. This (re)orientation ensures that AI-powered computational methods better reflect Indigenous intellectual traditions and uphold Indigenous Data Sovereignty (Carroll et al., 2020; Walter & Andersen, 2013; Warrior, 1994).

The ICA responds to the three structural challenges outlined earlier. First, it addresses epistemological misalignment by ensuring Indigenous theories of health and well-being guide model conceptualization and feature selection. Second, it confronts technical limitations by identifying where Indigenous determinants of health are absent from datasets. This is accomplished by structuring modeling decisions, so the computational workflow reflects Indigenous theoretical frameworks and community-defined priorities rather than defaulting to what is captured in clinical or administrative data. Third, the ICA embeds Indigenous governance processes by integrating Tribal Nation and Indigenous community oversight into the design, validation, and deployment of AI Models to uphold Indigenous Data Sovereignty.

**Fig. 2:**
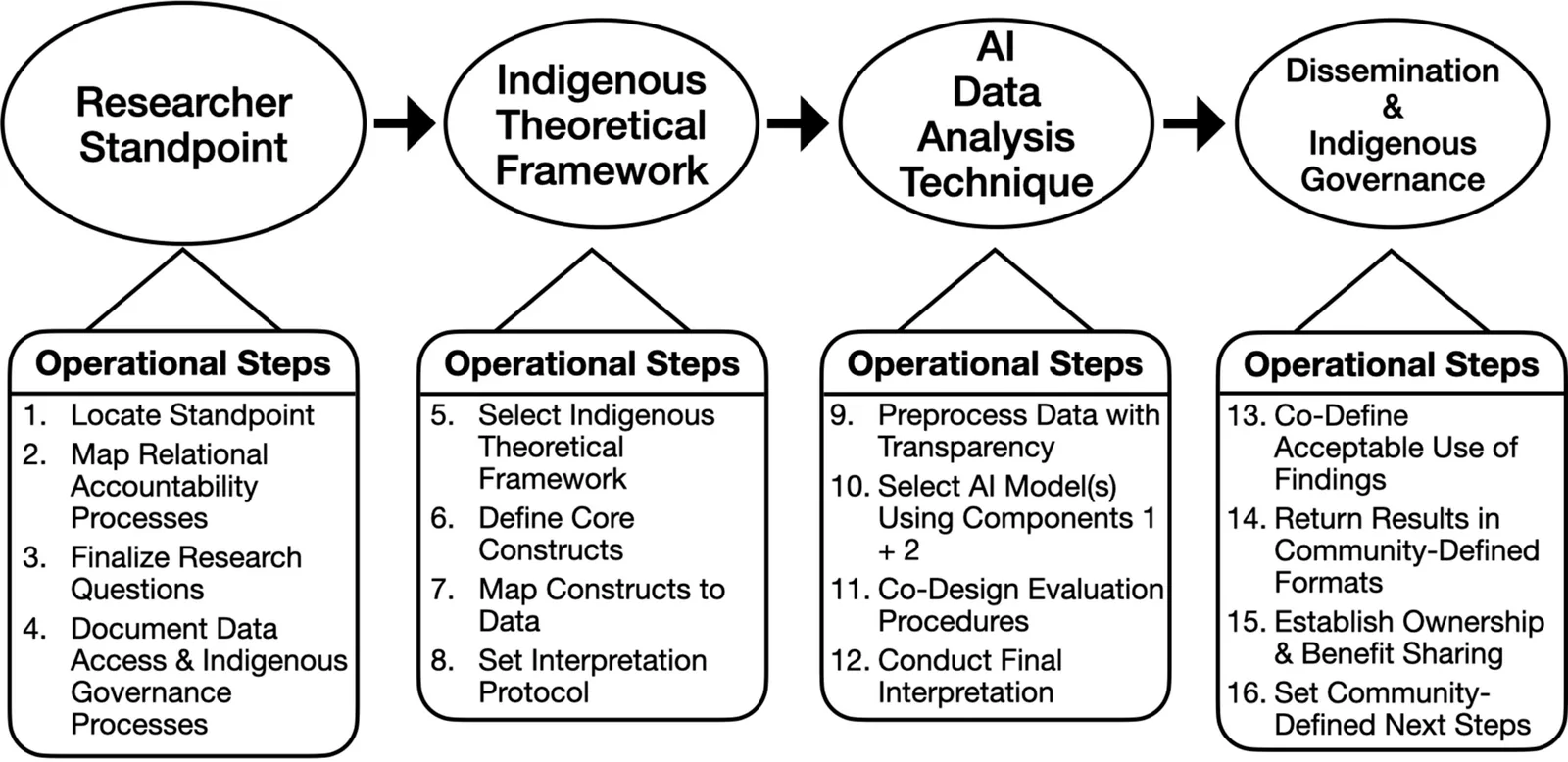
Components of the Indigenous Computational Approach with operation steps

The construction of Indigenous Statistical Spaces forms the core of the ICA. Building on previous work by Walter and Andersen (2013), the ICA comprises four interdependent components: (1) Researcher Standpoint; (2) Indigenous Theoretical Framework; (3) AI Data Analysis Technique; and (4) Dissemination and Indigenous Governance (see Fig. 2). Each component operationalizes an aspect of Indigenous Research Methodologies within AI-powered computational research by treating each component as interdependent elements rather than separate domains of inquiry. Taken together, the ICA provides a replicable protocol for building AI models that are culturally grounded or built from the “ground up” to be theoretically aligned and accountable to Indigenous populations (Okamoto et al., 2014).

The sections that follow elaborate each ICA component, in turn, articulating how Researcher Standpoint, Indigenous Theoretical Framework, AI Data Analysis Technique, and Dissemination and Indigenous Governance work together to construct Indigenous Statical Spaces for application in AI-powered prevention science research. Operational steps are provided for each component, along with reflection questions, to aid other researchers in applying the ICA for their own AI-powered prevention science research (see Table 1). This is also accompanied by the ICA Checklist (see Appendix). A case study on the Indigenous Wholistic Factors Project (IWFP) is included in this paper to demonstrate one application of the ICA in the context of suicide risk modeling to illustrate the ICA in practice.

**Table 1:** Reflection questions to guide the operational steps of the Indigenous Computational Approach

Reflection questions			
**Researcher Standpoint**	**Indigenous Theoretical Framework**	**AI Data Analysis Technique**	**Dissemination & Indigenous Governance**
How do my social identities (e.g., race, class, gender) shape my worldview and influence the knowledge I produce?	Which conventional theories shape my understanding of the social problem I am investigating?	How appropriate is the selected data analysis technique for understanding the social problem I am investigating?	How can results be shared in ways that are accessible and meaningful to the community?
What is my relationship to the Indigenous community I am working with?	What assumptions underlie these theories, and how do they reinforce or challenge colonial paradigms?	Does this AI method support Indigenous community participation and uphold Indigenous governance over interpretation and decision-making?	What role does the community play in determining how findings are interpreted and presented?
What is my relationship to the social problem I am investigating?	What Indigenous theories offer alternative frameworks for understanding this social problem?	Who determines which features are included, excluded, transformed, or prioritized?	What next steps does the Indigenous community view as best, and do mine differ?
Who has the authority to approve research question(s) and data usage?	How are Indigenous theoretical domains translated into measurable constructs?	How is model success defined?	
Where does my standpoint align with or diverge from the perspectives of the Indigenous community I am working with, and what ethical considerations arise from these differences?	What steps ensure that Indigenous perspectives are the primary lens of analysis across the computational workflow?		

### Component 1: Researcher Standpoint

Researcher Standpoint refers to the researcher’s social position or positionality as assembled by their lived experiences, epistemological commitments, and relationships to the Indigenous communities involved in the research (Hurley & Jackson, 2020). It, also, encompasses the researcher’s orientation to power and their responsibility to recognize and address power dynamics that may advantage some groups while marginalizing others (Muhammad et al., 2015). Within Indigenous Research Methodologies, standpoint determines how researchers enter into relationships with Indigenous communities (with whom they may or may not be a member or Tribal Citizen of), how they understand knowledge (where learning and unlearning may occur), and what obligations they carry across the life of a research project (whether research is extractive or participatory). Because Indigenous populations are diverse across Tribal Nations and geographies, locating standpoint also requires acknowledging where researcher standpoint aligns with or diverges from the potentially heterogeneous communities represented in the data. The ICA names these understandings and treats researcher standpoint as the first component in constructing an Indigenous Statistical Space.

Researcher standpoint informs the framing of the computational workflow by influencing what questions get asked, which constructs get privileged, how features are defined, what counts as evidence, and how model outputs are interpreted (Walter & Andersen, 2013). In Indigenous health research, these decisions are consequential because they determine whether prevention efforts reproduce biomedical assumptions or reflect Indigenous conceptions of well-being (Ansloos, 2018). For example, decision about feature inclusion or exclusion may unintentionally encode deficits-based views of Indigenous populations or overlook community-defined risk and protective factors in the computational workflow (Adams et al., 2024). This extends to how researchers understand and enact Indigenous Data Sovereignty. Decisions about data access and dissemination are inseparable from the commitments to Indigenous Data Sovereignty principles embedded in the researcher’s positionality and what they define as important.

Importantly, treating Researcher Standpoint as a procedural step does not imply that researcher identity is modeled as a statistical variable nor that interactions between researcher and participant identities are estimated quantitatively. Rather, this component structures the reflection on positionality and the documentation of how it can inform the computational workflow across data preprocessing steps, model development decisions, and interpretation procedures. This component’s purpose is to enhance transparency and interpretative accountability in decision-making processes. However, it does not replace established statistical procedures nor eliminate the need to attend to within-group heterogeneity in analytic design. By explicitly locating and documenting Researcher Standpoint at the onset, the ICA makes analytic assumptions visible to better align modeling decisions with community-defined priorities and Indigenous governance structures.

To support transparent and replicable application of the ICA, researchers are guided to locate and document their standpoint through a set of operational steps. Reflection questions that support this process are provided in Table 1 (see Table 1). In practice, articulating Researcher Standpoint involves:

1. **Locate Standpoint:** Name your positionality and describe how it shapes the research process.
2. **Map Relational Accountability Processes:** Identify governance partners (e.g., Community Advisory Board, Elders) who guide or approve research decisions.
3. **Finalize Research Question(s):** Work with governance partners to define or approve the research question(s).
4. **Document Data Access & Indigenous Governance Processes:** Describe Indigenous Data Sovereignty processes (e.g., Tribal IRB, data use agreement) required to acquire or generate and use data.

### Component 2: Indigenous Theoretical Framework

An Indigenous Theoretical Frameworks refers to the Indigenous Knowledge Systems that guide how health and well-being are conceptualized within a research project (Kovach, 2021). These frameworks are grounded in the bodies of intergenerational knowledge practices that are rooted in lived experiences, cultural traditions, and relational connections to land and community (Watson-Verran & Turnbull, 1995). Within Indigenous Research Methodologies, Indigenous Theoretical Frameworks commonly emphasize the interconnectedness of physical, mental, spiritual, and environmental health that are needed to foster holistic health and well-being (Absolon, 2010; Hart, 2010). Indigenous Theoretical Frameworks provide the conceptual foundation for defining what counts as a meaningful construct (what knowledge is valid), how risk and protection are understood (how knowledge is made legible), and which statistical associations are considered relevant (what insights are structured to be generated).

The selection of an Indigenous Theoretical Framework is critical because prevention research is never theory-neutral. In the absence of an Indigenous Theoretical Framework, AI-powered AI-powered prevention Science research can default to overlooking Indigenous determinants of health by omitting them in the computational workflow with the prioritization of mainstream, biomedical theories of risk (Friedman & Hansen, 2024; Walter & Andersen, 2013). These defaults can shape feature selection, feature construction, model selection, output interpretation, and even the definition of model success (Jacobs & Wallach, 2021; Obermeyer et al., 2019; Shmueli, 2010). As a result, AI models may achieve high statistical accuracy or generate novel insights, but still lack relevance or effectiveness in real-world Tribal healthcare settings.

To support transparent and replicable application of the ICA, researchers are guided to articulate their Indigenous Theoretical Framework through a set of operational steps. Reflection questions that support the selection of an appropriate theory are provided in Table 1 (see Table 1). In practice, documenting an Indigenous Theoretical Framework involves:

5. **Select the Indigenous Theoretical Framework:** Specify the Indigenous Theoretical Framework guiding the research process.
6. **Define Core Constructs:** Translate theoretical domains into measurable constructs.
7. **Map Constructs to Data:** Identify Indigenous-specific or community-defined features and name if constructs are missing.
8. **Select Interpretation Protocol:** Define how the selected Indigenous Theoretical Framework guides interpretation of model outputs and success.

### Component 3: AI Data Analysis Technique

AI Data Analysis Technique refers to the set of analytic decisions within the computational workflow that govern how data are prepared, modeled, evaluated, and interpreted. More specifically, this includes: data preprocessing steps, like data cleaning, feature selection and construction; model development decisions, like model or algorithm selection, validation and calibration strategies; and interpretation procedures, used to evaluate model outputs and statistical significance (Steyerberg & Vergouwe, 2014). Taken together, the AI Data Analysis Technique is the application of AI-powered computational methods to answer research questions. The ICA extends Indigenous Research Methodologies to include these quantitative and computational processes by linking analytic decisions to Researcher Standpoint and the selected Indigenous Theoretical Framework.

Although AI models themselves are often described as neutral tools, the analytic decisions that structure them are not (Jacobs & Wallach, 2021). Decisions about preprocessing, model development, and interpretation determine which forms of knowledge become visible, which statistical associations are amplified, and which patterns are rendered invisible (Passi & Barocas, 2019). When AI-powered computational methods are applied without grounding from Researcher Standpoint and Indigenous Theoretical Frameworks, AI models risk misrepresenting Indigenous health outcomes and reproducing extractive or deficits-based research practices (Lewis et al., 2024). For Indigenous populations, this misalignment can have direct consequences for how communities experience AI-powered prevention research.

To support transparent and replicable application of the ICA, researchers are guided to document their AI Data Analysis Technique through a set of operational steps. Reflection questions that support the analytic decision-making across the computation workflow are provided in Table 1 (see Table 1). In practice, applying AI Data Analysis Techniques involves:

9. **Preprocessing Data with Transparency:** Report harmonization, standardization, imputation, missing data handling, and governance partner-led preprocessing decisions.
10. **Select AI Model(s) Using Components 1 + 2:** Justify modeling choices (e.g., lasso, random forests, neural nets, etc.) based on Researcher Standpoint and Indigenous Theoretical Framework.
11. **Co-Design Evaluation Procedures:** Review model outputs including evaluation procedures (e.g., calibration, validation, other performance metrics) with governance partners.
12. **Conduct Final Interpretation:** Interpret all final model outputs using the selected Indigenous Theoretical Framework.

### Component 4: Dissemination and Indigenous Governance

Dissemination and Indigenous Governance refer to how research outputs are governed in their application and disseminated in ways that uphold Indigenous Data Sovereignty. Within the ICA, this component extends earlier work on Indigenous Statistical Spaces to address what happens after computational analysis (Walter & Andersen, 2013). This includes how results are returned to Indigenous communities, who has authority over their usage, and how research findings are translated into prevention efforts. The ICA treats dissemination and governance processes as an integral procedural component rather than a downstream or optional afterthought. Dissemination and Indigenous Governance operationalize the CARE Principles for Indigenous Data Governance by embedding them into the lifecycle of AI-powered prevention science research (Carroll et al., 2021). CARE stands for Collective Benefit, Authority to Control, Responsibility, and Ethics (Carroll et al., 2020). In practice, this means Indigenous communities are positioned to have active roles in how research outputs are applied and disseminated, rather than being passive recipients of the research process.

Dissemination and Indigenous Governance are critical because research outputs from AI-powered prevention science research can carry significant consequences for both, individuals and communities. Without proper governance, predictive risk models risk being misapplied in ways that conflict with Indigenous values or intensify experiences of surveillance and mistrust (Sierra et al., Under Review). Moreover, standard academic dissemination processes often fail to return knowledge to the communities from which data are derived (Israel et al., 1998; Tuck, 2009). By contrast, the ICA frames Dissemination and Indigenous Governance as essential processes that support Indigenous self-determination and ethical translation of evidence into prevention efforts.

To support transparent and replicable application of the ICA, researchers are guided to document their processes for Dissemination and Indigenous Governance through a set of operational steps. Reflection questions that support intentional Dissemination and Indigenous Governance are provided in Table 1 (see Table 1). In practice, applying this component involves:

13. **Co-Define Acceptable Use of Findings:** Specify guidelines from governance partners on how findings can be used, stored, or restricted.
14. **Return Results in Community-Defined Formats:** Determine how findings will be shared (e.g., Tribal council reports, community town halls, one-pagers, academic publications, etc.) and what cannot be shared.
15. **Establish Ownership & Benefit-Sharing:** Document agreements regarding authorship, intellectual property, and tangible benefits.
16. **Set Community-Defined Next Steps:** Include governance partner-defined recommended next steps and governance over model maintenance or discontinuation

## Case Study: Indigenous Wholistic Factor Project

This case study draws on the IWFP, a previously published empirical study that applied machine learning to predict suicidal ideation among Indigenous high school students in California (Sierra, 2025). Detailed descriptions of the dataset, feature selection and construction, computational modeling, validation procedures, and empirical findings are reported elsewhere (Sierra, 2025). The purpose of the present case study is not to replicate these results, but rather to illustrate in practice the operational steps of the ICA as used to construct an Indigenous Statistical Space and guide analytic decision-making in AI-powered prevention science research.

**Fig. 3:**
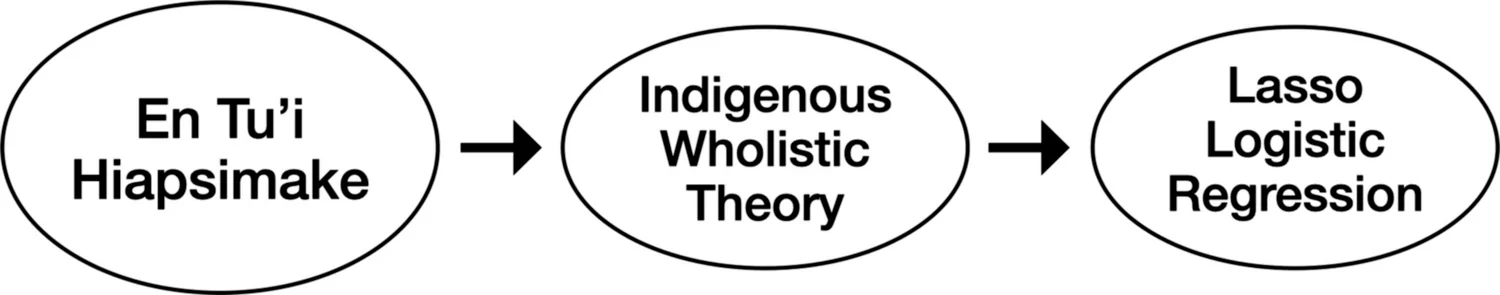
Constructed Indigenous Statistical Space for the Indigenous Wholistic Factors Project

The IWFP is highlighted as a case study for three reasons regarding the generalizability and applicability of demonstrating the ICA in practice: (1) use of a large, non-Tribal secondary dataset where Indigenous determinants of health are structurally constrained; (2) application of an AI-powered computational method commonly used in prevention science research; and (3) grounding in a Community-Based Participatory Research (CBPR) process that informed data preprocessing, model development, and interpretation procedures. These characteristics demonstrate how applying the ICA to construct Indigenous Statistical Spaces can be actualized even when data infrastructures are imperfect. As such, an Indigenous Statistical Space was constructed around the: (1) Researcher Standpoint of “en tui hiapsimake”; (2) Indigenous Theoretical Framework of Indigenous Wholistic Theory; and (3) AI-powered Data Analysis Technique of lasso logistic regression (see Fig. 3). The fourth component of the ICA is also discussed.

### Component 1: Researcher Standpoint—en tui’hiapsimake (“With Good Heart”)

The Researcher Standpoint for the IWFP was guided by my worldview as a Yo’eme (Yaqui) person, particularly the cultural value of en tui hiapsimake, translated as doing the work “with good heart” (Evers & Molina, 1987). My standpoint reflects the obligations that meaningful work must be carried with integrity through relational accountability and sustained commitment to community protocols. Rooted in the teachings of Yo’eme elders, knowledge, and by extension the generation of knowledge, is not extracted nor individually claimed, but rather entrusted and carried forward through reciprocal relationships and ceremonial responsibilities (Maaso et al., 1993). Additionally, human experiences are understood as inseparable from the land and the interconnected worlds that shape existence, which are not just physical places but spiritual and sacred spaces which require ethical engagement with both seen and unseen forces spanning across past, present, and future generations.

Within the ICA, the Research Standpoint of en tui hiapsimake shaped three commitments: (1) framing of the purpose of the research process as prevention-focused rather than diagnostic or punitive; (2) centering CBPR with Indigenous youth as primary knowledge holders of the social problem in their own lives; and (3) embedding ethical considerations governing the interpretation and dissemination of research outputs across all later components of the ICA. These commitments were operationalized through the ICA’s four Researcher Standpoint-related operational steps. Step 1 (Locate Standpoint) was enacted by explicitly naming my Yo’eme identity and how the cultural value of en tui hiapsimake shaped my motivations for conducting research by, with, and for diverse, Intertribal Indigenous young people. Step 2 (Map Relational Accountability Processes) involved identifying the Sacramento Native American Health Center, an Urban Indian Health Organization in Sacramento, California, and its Native Youth Ambassadors Program as governance partners to anchor the project in Indigenous-led decision-making and CBPR processes. Step 3 (Finalize Research Questions) was initiated by the Indigenous young people, themselves, whose questions about using AI-powered computational methods to understand the risk and protective factors of suicidal ideation among Indigenous youth in California directly motivated the IWFP. With this, members of the Native Youth Ambassadors Programs defined and approved the final research question. Step 4 (Document Data Access & Indigenous Governance Processes) was fulfilled by leadership support from the Sacramento Native American Health Center who approved the project scope and the use of secondary data from the California Department of Education. The California Healthy Kids Survey was selected to maximize the available sample of Indigenous high school students and enable a population-level, or as the youth described it, a statewide “big picture” investigation across a heterogeneous and diverse, Intertribal sample rather than a single Tribe-specific inquiry.

### Component 2: Indigenous Theoretical Framework—Indigenous Wholistic Theory

The IWFP was guided by the Indigenous Wholistic Theory, a theoretical framework that centers relational, intergenerational, and community-defined perspectives on well-being (Absolon, 2010). Indigenous Wholistic Theory conceptualized health as a multifaceted integration of individual, spiritual, emotional, mental, and physical domains that are each embedded within and shaped by historical, social, political, and economic contexts. Within this framework, well-being emerges when these domains and contexts are in balance, while outcomes such as suicidal ideation can result from disruptions across any of these interconnected dimensions. This theoretical framework was selected by the youth in part because of its Intertribal applicability to allow for conceptual grounding in a diverse, heterogenous Indigenous sample without imposing a single Tribe-specific theoretical viewpoint.

Applied within the ICA, Indigenous Wholistic Theory functioned as a structuring framework that governed how risk and protective factors were conceptualized, grouped, and interpreted. This theoretical framework motivated moving beyond a psycho-centric conceptualization of suicide-related behavior which often overemphasize individual-level, clinical risk factors (Ansloos, 2018; White, 2017). Instead, Indigenous Wholistic Theory situated the selection of features within a more wholistic understanding of how multiple domains interact to shape the well-being of Indigenous youth. This Indigenous Theoretical Framework expanded the analytic focus beyond a singular focus on individual-level, clinical risk factors to include features across five conceptual domains from the Indigenous Wholistic Theory including (1) individual; (2) spiritual-historical; (3) emotional-social; (4) mental-political; and (5) physical-economic. This shift allowed for a more comprehensive and culturally grounded analysis that reflects the lived realities of Indigenous high school students in California.

This process was operationalized through the ICA’s four Indigenous Theoretical Framework Standpoint-related operational steps. Step 5 (Select the Indigenous Theoretical Framework) was achieved through review and approval of the Indigenous Wholistic Theory by the members of the Native Youth Ambassadors program who affirmed its relevance for understanding well-being among Indigenous youth across diverse, Intertribal contexts. Step 6 (Define Core Constructs) translated the five conceptual domains of the Indigenous Wholistic Theory into analytically defined categories that structured both feature selection and interpretation. Step 7 (Map Constructs to Data) involved assessing the extent to which available features in the dataset aligned with each conceptual domain, as well as documenting domain-level absences. First, this step was carried out through a CBPR activity with the members of the Native Youth Ambassadors program, who reviewed the full set of available variables and determined which features were most relevant to understanding suicidal ideation from their perspective. The youth then organized their selected features into the five conceptual domains based on their understanding of well-being and the guiding theoretical framework. Lastly, this process included explicit discussion and documentation of the absence of variables relevant to the spiritual-historical domain such as land-based relationships and culturally specific identity practices central to the Indigenous Wholistic Theory (Sierra, 2025). Accordingly, the present analysis represents a partial operationalization of the selected theoretical framework due to the structural constraints of the dataset. Step 8 (Set Interpretation Protocol) established that retained features would be interpreted in relation to their domain-level meaning rather than as isolated individual risk markers. For example, breakfast consumption was interpreted as an indicator of physical-economic stability rather than personal behavior alone. Importantly, Indigenous Wholistic Theory structured the conceptual organization of the youth-selected factors and their domain-level interpretation to ensure that model outputs were understood within their broader contexts instead of as isolated statistical associations.

### Component 3: AI Data Analysis Technique—Machine Learning with Lasso Logistic Regression

The IWFP employed a machine learning algorithmic approach using lasso logistic regression to identify the most significant features for predicting suicidal ideation among Indigenous high school students in California (Breiman, 2001). The study used cross-sectional data from the 2019–2020 California Healthy Kids Survey which included a subset of self-identified Indigenous high school students (*n* = 2609). The sample represents youth from multiple Tribal Nations and urban contexts across California to reflect the youth-driven statewide “big picture” investigation aimed at understanding suicidal ideation across diverse, Intertribal populations. As a limitation, Tt sample does not estimate Tribe-specific variation within the aggregated dataset. The outcome variable was the presence of suicidal ideation measured using the self-report item: “During the last 12 months, did you ever seriously consider attempting suicide?”.

Lasso logistic regression with an L1 penalty was used to select the most significant features that predict the outcome of suicidal ideation from a set of 17 candidate features selected by the members of the Native Youth Ambassadors program during Step 7. Lasso logistic regression shrinks the coefficients of the least influential features to zero, effectively removing them from the model to retain the strongest subset of features (Hastie et al., 2016). The tuning parameter lambda was optimized using tenfold cross-validation, which balances coefficient shrinkage while providing stable performance estimates of predictive performance in new cases (Steyerberg & Vergouwe, 2014). This approach reduces overfitting and ensures model performance beyond the training set. Model discrimination was assessed using the area under the receiver operating characteristic curve (AUC) and interpreted using standard thresholds (Hosmer et al., 2013). Calibration was evaluated using the Brier score to assess the agreement between predicted and observed outcomes (Brier, 1950).

Using lasso logistic regression with a mean lambda of 0.0078412, the model retained ten out of the 17 candidate features as predictors of suicidal ideation among Indigenous high school students in California. These included: depressive symptoms; school-based victimization; sexual and gender minority status; lifetime use of alcohol, vapes, and cannabis; breakfast consumption; access to alcohol and other drugs; and parent education level. Following tenfold cross-validation, the final model achieved a cross-validated mean AUC of 0.87 (BC 95% CI 0.84–0.88), indicating strong discriminatory performance within the sample (Sierra, 2025). The Brier score of 0.10 indicated strong agreement between predicted and observed outcomes, meeting standard benchmarks for model calibration.

These analytic decision and outcomes were achieved through the ICA’s four AI Data Analysis Technique-related operational steps. Step 9 (Preprocess Data with Transparency) involved restricting the analytic sample to self-identified Indigenous participants, standardizing continuous variables, and conducting sensitivity analyses to assess the impact of missing data. Step 10 (Select AI Model Using Components 1 + 2) motivated the selection of lasso logistic regression because the youth-developed research question required identifying a parsimonious and interpretable subset of predictors within a moderately high-dimensional dataset while minimizing overfitting. Additionally, lasso logistic regression was selected and approved by the members of the Native Youth Ambassadors program to account for statistical considerations concerning multicollinearity management and interpretability. In other words, this allowed for the retained features to be more meaningfully situated and interpreted within the conceptual domains defined by the Indigenous Wholistic Theory. The AI model and statistical method were selected to maximize the alignment between the selected framework and the  technical appropriateness of the model, rather than being solely driven by the performance or convenient properties inherent to the model itself.

Step 11 (Co-Design Evaluation Procedures) guided the CBPR processes where members of the Native Youth Ambassadors program co-interpreted standard model evaluation metrics including discrimination, calibration, and overall accuracy. Step 12 (Conduct Final Interpretations) required that all model outputs be interpreted in relation to their domain-level meaning and reviewed with the youth governance partners. Of note, these discussions with members of the Native Youth Ambassadors program addressed the acceptability of model error to prioritize false positive over false negative in supporting early connection to care for Indigenous youth.

### Component 4: Dissemination and Indigenous Governance

Dissemination and governance were treated as integral components of the computational workflow rather than post hoc activities. Findings from the IWFP were returned to Indigenous high school students and community partners through town halls and community gatherings held across Northern California in collaboration with various Urban Indian Health Organizations. These activities emphasized the accessible and widespread dissemination of the findings. Additionally, these activities were oriented to inform future, community-led suicide prevention efforts for urban Indigenous youth in California. A community-identified next step included the subsequent development of a culture-based suicide risk assessment tool for use at the Sacramento Native American Health Center (Sierra, 2023b).

Dissemination decisions were guided by the CARE Principle of Collective Benefit and the prioritization of formats that support shared understanding and community use rather than academic dissemination alone (Carroll et al., 2020, 2021). Under the CARE Principle of Authority to Control, decisions regarding study documentation and replicable code sharing were made following community discussions about appropriate use, reuse, and adaption. Replicable documentation and code scripts were made publicly available only after these discussions with the intention of supporting other Indigenous communities who may wish to adapt and implement similar AI-powered prevention science research (Sierra, 2023a).

These activities were guided by the ICA’s four Dissemination & Indigenous Governance-related operational steps. Step 13 (Co-Define Acceptable Use of Findings) involved discussions with leadership from the Sacramento Native American Health Center and its Native Youth Ambassadors program members to clarify how model outputs could and could not be used. Step 14 (Return Results in Community-Defined Formats) guided the use of town halls and community gatherings, instead of relying on academic publishing alone. Step 15 (Establish Ownership and Benefit-Sharing) documented agreements regarding authorship for academic publishing and capacity-building for Indigenous youth to learn about AI-powered prevention science research. This also included the release of publicly available documentation to support adaption of these processes by other Indigenous communities. Step 16 (Set Community-Defined Next Steps) resulted in community-led recommendations for future work including the development of a culture-based risk assessment tool.

## Discussion

This paper presents the ICA as a structured reflexive protocol for advancing prevention science research by integrating Indigenous Research Methodologies with AI-powered computational methods. By formalizing the construction of Indigenous Statistical Spaces, the ICA reorients how AI models are designed, validated, and deployed for prevention science research to better ensure that it aligns with Indigenous determinants of health and community-defined priorities. This reframing is particularly critical in addressing deaths of despair among Indigenous populations, where AI models are constrained by epistemological, technical, and ethical challenges. This paper articulates a replicable set of operational steps to structure the computational workflow within the ICA. Specifically, the ICA structures four interdependent components: (1) Researcher Standpoint; (2) Indigenous Theoretical Framework; (3) AI Data Analysis Technique; and (4) Dissemination and Indigenous Governance. These components are supported by a series of operational steps and the ICA Checklist (Appendix) to aid other researchers in applying the ICA for their AI-powered prevention science research.

Additionally, the case study on the IWFP demonstrates how the ICA functions in practice. Several retained features including depressive symptoms, school-based victimization, and substance use are consistent with prior research identifying relevant correlates of suicidal ideation among Indigenous youth (Fetter et al., 2023; Wiglesworth et al., 2022). However, the identification of sexual and gender minority status underscores the importance of attending to intersecting identities in Indigenous youth mental health research. This is an area that remains comparatively underexamined in Indigenous research and statewide population-level datasets (Green et al., 2022).

Taken together, the findings align with established evidence regarding the multidimensional nature of suicide risk among Indigenous adolescents while extending attention to intersectional identities for Indigenous and sexual and gender minority youth within aggregated datasets. The primary contribution of this case study lies not in the identification of specific or novel predictors rather, the contribution lies in the structured integration of Indigenous Research Methodologies within a computational workflow that prioritized Indigenous theoretical framing, youth-governed feature selection, transparent documentation of analytic decision-making, and explicit acknowledgment of structural measurement gaps in a large, non-Tribal secondary dataset. The ICA demonstrates how conventional AI models and statistical methods can be embedded within a community-driven and reflexively documented analytic process without sacrificing empirical rigor.

The ICA does not claim to automatically produce different empirical findings or superior model accuracy. Rather, it alters the conditions under which AI-powered computational methods are designed, validated, and deployed across the computational lifecycle. The ICA posits that when AI models are culturally grounded or built from the ground up to be aligned and accountable to Indigenous populations, they can more transparently represent Indigenous determinants of health and support Indigenous Data Sovereignty. As AI continues to shape prevention science, the central question is no longer whether AI will be used in Indigenous health research, but under whose terms and with what governance partnerships. The ICA offers a replicable set of operational steps for ensuring AI-powered prevention science research is accountable to Indigenous communities. Future research should build on this foundation by applying and expanding this protocol through Indigenous-led development of theoretical constructs and frameworks, expansion of culturally grounded measurement, and systematic evaluation of the use of AI models across diverse, Intertribal and Tribe-specific datasets. By doing so, prevention science can advance more transparent and culturally responsive computational practices that support Indigenous health and self-determination.

## Supplementary Information

Below is the link to the electronic supplementary material.

ESM 1 (DOCX 19.4 KB)

## Data Availability

The study documentation and replicable code for the case study mentioned in this manuscript are available at https://osf.io/4dpwt/ following community discussions to ensure alignment with Indigenous Data Sovereignty principles.

## Declarations

### Clinical Trial Number

Clinical trial number: not applicable.
