# Supplementary material for: A Guide to Constructing Indigenous Statistical Spaces for Prevention Science Research
Source: Prev Sci. 2026 May 8;27(7):1239–52. doi: 10.1007/s11121-026-01911-5 (PMC13189265; doi:10.1007/s11121-026-01911-5)
Supplement: Supplementary file 1 [file 11121_2026_1911_MOESM1_ESM.docx]

**Appendix A: Indigenous Computational Approach Checklist**

| **Step #** | **Guide Question** | **Page Addressed** |
| --- | --- | --- |
| **Component 1: Researcher Standpoint** | | |
| 1 | **Is the researcher’s positionality clearly described?** (e.g., social identities, relationship to the community, relationship to the social problem) |  |
| 2 | **Are relational accountability processes and governance partners described and identified?** (e.g., community advisory board with shared decision-making and iterative feedback processes) |  |
| 3 | **Have the governance partners been involved in defining or approving the research question(s)?** |  |
| 4 | **Are data access and Indigenous governance processes documented?** (e.g., Tribal IRB approval, data sharing agreements) |  |
| **Component 2: Indigenous Theoretical Framework** | | |
| 5 | **Is an Indigenous Theoretical Framework explicitly identified and named?** |  |
| 6 | **Are core constructs defined and derived from the selected framework, rather than imported from a mainstream theory without critical examination for misalignment or colonial assumptions?** |  |
| 7 | **Are Indigenous-specific or community-defined features meaningfully incorporated into the model, including acknowledgement of missing constructs where applicable?** (e.g., through feature selection with inclusion or exclusion justification) |  |
| 8 | **Is there a theory-driven protocol for interpreting model outputs and defining success?** |  |
| **Component 3: AI Data Analysis Technique** | | |
| 9 | **Are data preprocessing steps reported, including documentation of any governance partner-led preprocessing decisions where applicable?** (e.g., harmonization, standardization, imputation, missing data handling) |  |
| 10 | **Are modeling choices justified with reference to Components 1 and 2, rather than relying solely on statistical performance or convenience?** |  |
| 11 | **Are evaluation procedures co-designed or reviewed with governance partners?** (e.g., validation, calibration, other performance metrics) |  |
| 12 | **Are final model outputs interpreted using the selected Indigenous Theoretical Framework?** |  |
| **Component 4: Dissemination and Indigenous Governance** | | |
| 13 | **Have the governance partners defined acceptable uses or restrictions for findings?** |  |
| 14 | **Are results returned to the community in accessible and appropriate formats as defined by the governance partners?** (e.g. Tribal council reports, community town halls, academic publications) |  |
| 15 | **Are agreements regarding authorship, ownership, and intellectual property documented, including tangible or reciprocal benefits to the community?** |  |
| 16 | **Are community-defined next steps and governance over model maintenance or discontinuation specified?** |  |
