# Supplementary material for: A Guide to Constructing Indigenous Statistical Spaces for Prevention Science Research
Source: Prev Sci. Author manuscript; Available in PMC 2026 May 20. (PMC13189265; doi:10.1007/s11121-026-01911-5)
Supplement: Appendix [file NIHMS2174660-supplement-Appendix.pdf]

Appendix A: Indigenous Computational Approach Checklist

Step #	Guide Question	Page Addressed
**Component 1: Researcher Standpoint**		
1	**Is the researcher's positionality clearly described?** (e.g., social identities, relationship to the community, relationship to the social problem)	
2	**Are relational accountability processes and governance partners described and identified?** (e.g., community advisory board with shared decision-making and iterative feedback processes)	
3	**Have the governance partners been involved in defining or approving the research question(s)?**	
4	**Are data access and Indigenous governance processes documented?** (e.g., Tribal IRB approval, data sharing agreements)	
**Component 2: Indigenous Theoretical Framework**		
5	**Is an Indigenous Theoretical Framework explicitly identified and named?**	
6	**Are core constructs defined and derived from the selected framework, rather than imported from a mainstream theory without critical examination for misalignment or colonial assumptions?**	
7	**Are Indigenous-specific or community-defined features meaningfully incorporated into the model, including acknowledgement of missing constructs where applicable?** (e.g., through feature selection with inclusion or exclusion justification)	
8	**Is there a theory-driven protocol for interpreting model outputs and defining success?**	
**Component 3: AI Data Analysis Technique**		
9	**Are data preprocessing steps reported, including documentation of any governance partner-led preprocessing decisions where applicable?** (e.g., harmonization, standardization, imputation, missing data handling)	
10	**Are modeling choices justified with reference to Components 1 and 2, rather than relying solely on statistical performance or convenience?**	
11	**Are evaluation procedures co-designed or reviewed with governance partners?** (e.g., validation, calibration, other performance metrics)	
12	**Are final model outputs interpreted using the selected Indigenous Theoretical Framework?**	
**Component 4: Dissemination and Indigenous Governance**		
13	**Have the governance partners defined acceptable uses or restrictions for findings?**	
14	**Are results returned to the community in accessible and appropriate formats as defined by the governance partners?** (e.g. Tribal council reports, community town halls, academic publications)	
15	**Are agreements regarding authorship, ownership, and intellectual property documented, including tangible or reciprocal benefits to the community?**	
16	**Are community-defined next steps and governance over model maintenance or discontinuation specified?**	
